# The impact of smartphone addiction on mental health and its relationship with life satisfaction in the post-COVID-19 era

**DOI:** 10.3389/fpsyt.2025.1542040

**Published:** 2025-03-18

**Authors:** Chunyue Zhu, Shuo Li, Lei Zhang

**Affiliations:** ^1^ School of Marxism, Chuzhou University, Chuzhou, China; ^2^ School of Civil and Architecture Engineering, Chuzhou University, Chuzhou, China; ^3^ School of Earth and Environment, Anhui University of Science & Technology, Huainan, China

**Keywords:** smartphone addiction, negative emotions, depression, health care, COVID-19

## Abstract

Following the COVID-19 outbreak, smartphone usage among quarantined Chinese college students surged significantly, leading to a growing dependency on their devices and adversely impacting their emotional well-being. Consequently, the negative emotions associated with smartphone addiction have emerged as critical factors influencing students’ life satisfaction. However, the psychological mechanisms linking these emotional effects to life satisfaction, as well as potential sustainable educational interventions, remain insufficiently explored. This study assessed a sample of 322 undergraduates (51.6% male) using the Mobile Phone Addiction Index (MPAI), the Depression, Anxiety, and Stress Scale (DASS-21), and the Satisfaction With Life Scale (SWLS) to construct a mediation model. The aim was to examine the relationships between smartphone addiction, negative emotions, and life satisfaction. Results revealed that smartphone addiction was significantly positively correlated with negative emotions (r = 0.332, p < 0.01), while negative emotions were significantly negatively correlated with life satisfaction (r = -0.391, p < 0.01). Among these negative emotions, depression emerged as a key factor, intensifying smartphone dependence and detrimentally affecting overall life satisfaction. The mediation analysis demonstrated that smartphone addiction contributes to the development of negative emotions, which in turn reduce life satisfaction. Students with higher levels of smartphone addiction reported heightened negative emotions, leading to more pessimistic coping strategies and, ultimately, a decline in mental health and life satisfaction. This study provides valuable insights into the impact of smartphone addiction on college students’ life satisfaction and offers actionable educational recommendations to mitigate its harmful effects, promoting healthier and more sustainable lifestyles for students.

## 1 Introduction

Smartphones are widely used around the world and have become an indispensable part of our daily lives (1). However, with the proliferation of smartphones, the issue of smartphone addiction has become increasingly prominent (2). Recent studies have shown that smartphone addiction is related to both personal psychological factors and environmental factors (3). Personal psychological factors include anxiety, loneliness, and stress (4), all of which, when accumulated over time, can lead individuals to use their phones frequently as a way to escape from the real world (5). At the same time, environmental factors also play a significant role, particularly since smartphone applications and social media, by providing mobile and social functionalities, make it even harder for users to detach from their phones (6). Additionally, habitual smartphone use is an important factor contributing to smartphone addiction (7, 8). Specifically, habitual phone use not only reduces opportunities for face-to-face interactions (9), but can also lead to issues such as attention dispersion (10) and reduced sleep time (11), which in turn negatively impacts mental health (12). Since the outbreak of COVID-19 in early 2020, the pandemic has rapidly evolved into a global public health crisis (13). To control the spread of the virus, college students have had to adapt to a series of new lifestyles (14), including widespread adoption of online education (15), suspension of outdoor activities, and strict adherence to social distancing measures in public places (16). Recent research indicates that college students have used their phones excessively more frequently compared to before the outbreak (17). Consequently, during the pandemic lockdown, smartphones have become central to college students’ daily lives, greatly facilitating communication and information access, while also increasing the risk of smartphone addiction.

The sociodemographic characteristics, such as gender and age, also play a significant role in smartphone addiction (18). Notably, there are significant differences between men and women in their smartphone usage habits and addiction tendencies (19). Men tend to use smartphones more for gaming and social media, whereas women are more inclined to use them for social interaction and communication (20). The differences in usage motivations may lead to varying risks of addiction (21). Correspondingly, the anxiety experienced by women during social interactions may make them more susceptible to smartphone addiction (22), while men are more likely to become addicted due to immersion in the virtual world (23). Additionally, women may be more inclined to use their smartphones to maintain emotional connections with others, while men more often use their smartphones to escape from stress (24). Therefore, the performance of addictive behaviors by different genders may have different effects on the mental health and life satisfaction of individuals.

Some scholars have proposed that smartphone addiction can lead to negative impacts (25). For instance, prolonged immersion in the smartphone can trap individuals in a virtual world, causing them to overlook the beauty of real-life experiences and interpersonal relationships (26). Research indicates that smartphone addiction also affects college students’ physical and mental health, significantly impacting their physiological well-being, psychological state, and social interactions (27). Extended smartphone use not only puts considerable strain on students’ eyesight but also frequently leads to health issues such as cervical spine problems (28). Moreover, the negative emotions such as loneliness, anxiety, and depression resulting from smartphone addiction are becoming increasingly prominent, severely disrupting the psychological well-being of college students (29). Additionally, this addiction can erode students’ social interactions, reducing their engagement with the real world, affecting their interpersonal skills and academic performance, and even posing potential threats to their future career planning and development (30). Therefore, the increase in negative emotions due to changes in psychological state may affect college students’ life satisfaction.

Anxiety, depression, and stress levels, as negative emotional states, are key indicators that affect mental health (31). Previous research has shown that increases in these indicators can lead to worsened mental health (32), physical impairment and cognitive decline (33), and an increased risk of suicide (34). Particularly for college students, negative emotions are more prone to fluctuations under multiple stresses, leading to academic and social difficulties, and reducing life satisfaction and well-being (35). Factors such as academic and work-related stress, socioeconomic conditions (e.g., social risks), and environmental factors (e.g., air quality index) can exacerbate anxiety, resulting in various health problems (36), such as insomnia (37) and accelerated heart rate (38). Furthermore, depression, as a serious mental health condition, can significantly impact an individual’s emotions, cognition, and daily functioning (39). Work-related stress, job burnout, and lack of social support can all intensify depressive symptoms (40). In recent years, with the increasing stresses of academic and personal life, college students have become a high-risk group for this mental illness (39). Depression among college students can lead to social avoidance, decreased academic or work performance, and increased suicide risk, thereby severely affecting personal quality of life (40). At the same time, stress, as a physical and psychological response to a difficult environment, is primarily triggered by factors such as long working hours, moral dilemmas, and traumatic events (41). Chronic stress not only potentially leads to physical health issues, including muscle tension, respiratory problems, and serious diseases, but can also provoke emotional problems such as anxiety and depression (42). Therefore, anxiety, depression, and stress further exacerbate the psychological and physical burden on individuals, having a profound negative impact on college students’ health.

There may be a significant relationship between negative emotions such as depression, anxiety, and stress, and smartphone addiction. Individuals with high levels of depression often feel uncomfortable with face-to-face interactions and face more interpersonal difficulties, leading to a decrease in their life satisfaction (43). This indicates that an increased risk of smartphone addiction reduces positive emotional experiences, thereby affecting individual life satisfaction. Furthermore, negative emotions can be considered a mediating variable between smartphone addiction and life satisfaction. Individuals experiencing negative emotional states such as anxiety, depression, or loneliness may increase their smartphone usage as a strategy for emotional regulation (44). By focusing on negative emotions as a mediating variable, we can gain insight into the driving forces behind this behavior, rather than merely observing the frequency of mobile phone use. For example, research indicates that individuals in a low mood are more likely to seek support through social networks, often overlooking the potential risk of addiction (45). This suggests that emotional states significantly influence not only the behavior of smartphone usage but also the choices made in the context of social interactions online (46). Thus, understanding the complex relationship between emotional wellbeing and mobile technology use is imperative for addressing potential negative outcomes associated with excessive smartphone reliance.

Therefore, this study will focus on three aspects: (1) explaining the relationship between gender characteristics, habitual usage factors, and smartphone addiction; (2) assessing the role of negative emotions as a mediating variable; (3) revealing the mechanism by which negative emotions affect life satisfaction.

## 2 Materials and methods

### 2.1 Study design and participant recruitment

From March to April 2024, an electronic survey was conducted through the Chinese online questionnaire platform “Wenjuanxing”. The participants of this study were mainly full-time undergraduate students of Chuzhou University. All participants were informed about the purpose and procedures of the research, and the researchers could not access the students’ names through their ID numbers. In addition, the researchers ensured the confidentiality of the data.

### 2.2 Demographic and social information

In this study, there were 166 male participants (51.6%) and 156 female participants (48.4%) (**Table 1**). Regarding family background, 222 students (68.9%) were not only children, while 100 students (31.1%) were only children. Concerning economic status, 83 students (25.8%) were classified as economically disadvantaged, whereas 239 students (74.2%) were not identified as economically disadvantaged.

**Table 1:** The sociological characteristics of the population are the factors of habitual mobile phone use(N=322).

		N	%
Gender	Male	166	51.6
	Female	156	48.4
Grader	Freshman	262	81.4
	Sophomore	60	18.6
Whether it is an only child	No	222	68.9
	Yes	100	31.1
Live in difficulty	No	83	25.8
	Yes	239	74.2
How much time do you use your mobile phone every day	Less than 2 hours	13	4.0
	2-4 hours	41	12.7
	4-6 hours	103	32.0
	6-8 hours	91	28.3
	More than 8 hours	74	23.0
Use your phone regularly	00:00-6:00	11	3.4
	6:00-12:00	15	4.7
	12:00-18:00	74	23.0
	18:00-24:00	222	68.9

Additionally, 222 students (68.9%) reported peak mobile phone use between 18:00 and 24:00, 74 students (23.0%) reported peak use between 12:00 and 18:00, 15 students (4.7%) reported peak use between 06:00 and 12:00, and 11 students (3.4%) reported peak use between 00:00 and 06:00.

MPAI (Mobile Phone Addiction Index) (47), which comprises 17 items, evaluates four key factors: Impulse Control (the inability to regulate excessive phone use), Withdrawal Symptoms (negative emotional responses when access to the phone is restricted), Escapism (using the phone to avoid real-life problems), and Inefficiency (disruption of daily learning and work efficiency due to excessive phone use). Participants rate their responses on a scale from 1 (never) to 5 (always), with higher scores indicating a greater tendency toward mobile phone addiction. The total score ranges from 17 to 85, with three operationally defined diagnostic thresholds. Specifically, the scale classified individuals scoring between 17 and 30 points as non-addicted, those scoring between 30 and 50 points as mildly addicted, and participants scoring between 51 and 85 points as severely addicted. This hierarchical classification system was established based on the fundamental principle that higher total scores reflect more pronounced levels of addiction severity, with a Cronbach’s alpha coefficient of 0.897.

DASS-21 (Depression, Anxiety, and Stress Scale) (48) is a psychological instrument designed to evaluate an individual’s levels of depression, anxiety, and stress over a specified period. Depression is characterized by anhedonia and negative emotions, while anxiety is marked by excessive physiological arousal. Both conditions commonly involve pervasive stress experiences (49). Responses are rated on a 4-point scale: 1 (not at all), 2 (sometimes), 3 (often), and 4 (always), reflecting the intensity of depression, anxiety, and stress symptoms. The raw scores of each subscale are transformed by multiplying by a factor of 2 to yield the standardized subscale scores, where elevated scores reflect increased severity of the corresponding psychological construct. Regarding the depression subscale, the established clinical cutoffs for mild, moderate, and severe depressive symptoms are 24, 28, and 35 points, respectively. Parallel scoring thresholds have been validated for the anxiety subscale, with scores of 22, 24, and 29 points representing mild, moderate, and severe anxiety symptoms, respectively. Correspondingly, the stress subscale utilizes cutoff scores of 29, 33, and 40 points to categorize individuals into mild, moderate, and severe stress levels, respectively. The scale demonstrates high internal consistency, with a Cronbach’s alpha coefficient of 0.951.

SWLS (Satisfaction With Life Scale) is utilized to assess an individual’s overall life satisfaction, serving as a measure of subjective well-being. Life satisfaction is closely related to the concept of quality of life, focusing on the psychological significance of one’s standard of living. According to Pavot and Diener, life satisfaction encompasses a broad perspective that accounts for varying standards of satisfaction experienced by individuals across different times and locations (50). Other scholars describe life satisfaction as a subjective evaluation of one’s overall contentment with life. The scale comprises 5 items, with responses rated on a 7-point scale: 1 (strongly disagree), 2 (disagree), 3 (somewhat disagree), 4 (neutral), 5 (somewhat agree), 6 (agree), and 7 (strongly agree). The scale exhibits strong internal consistency, with a Cronbach’s alpha coefficient of 0.860.

### 2.3 Data analysis methods

This study employed SPSS 25 and the R programming language for data analysis. SPSS was primarily utilized for preliminary data processing, descriptive statistics, reliability and validity testing, and correlation analysis (51). Additionally, the SPSS Process macro plugin was used for mediation analysis. Specifically, PROCESS macro version 3.5, model 4, was applied to test the proposed mediation effects, with bootstrap iterations set to 500. Moderation effects were assessed using PROCESS macro version 3.5, model 1. Path analysis to evaluate the mediation model and its validity was conducted in SPSS. Box plots were created using the R programming language.

## 3 Results

### 3.1 Simple analysis of MPAI scale and DASS scale and descriptive statistics of some problems of depression scale

**Figure 1:**
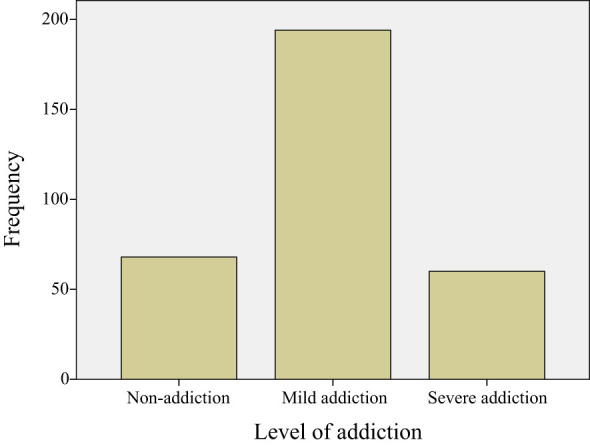
Mobile phone addiction grade frequency table (N=322). A score greater than 30 is considered to have the characteristics of mobile phone addiction, including mild and toxic mobile phone addiction.

This study revealed that a majority of participants 60.2% exhibited mild mobile phone addiction, while 18.6% showed severe addiction, and only 21.2% were identified with non- addiction. As shown in **Figure 1**, the sample with the characteristics of mobile phone addiction accounted for 78.8%. We tested the Spearman correlation between addicted and non-addicted samples and negative emotions respectively. The study found that samples with addiction had a positive effect on negative emotions, while samples without addiction had lower negative emotional feedback (**Table 2**).

**Table 2:** Spearman phase relation table.

	Number of cases	Spearman correlation coefficient		
		Depression	Anxiety	Stress
Have a sample of mobile phone addiction	254	0.175**	0.269**	0.280**
There was no sample of phone addiction	68	0.228	0.121	0.147

**p<0.01.

According to the prevalence of psychological dimension assessed by DASS-21 scale, the specific data showed that the prevalence of depressive symptoms was 3.9% for mild symptoms, 2.5% for moderate symptoms, and 1.1% for severe symptoms. The prevalence of anxiety symptoms was mild 34.7%, moderate 16.5%, severe 23.2%; The prevalence of stress symptoms was mild 21.4%, moderate 13.7%, and severe 3.5%.

The results presented in **Table 3** indicate that issues such as anhedonia (inability to experience pleasure), difficulty initiating tasks, lack of hope for the future, feelings of sadness and depression, and lack of enthusiasm each have a prevalence exceeding 50%. Additionally, concerns regarding feelings of worthlessness and perceiving life as meaningless are evident in approximately 25% of the cases. These findings suggest that the phenomenon of depression is notably severe among the study participants.

**Table 3:** Percentage situation(N=322).

	Not conforming	Sometimes agree	Often agree	Always agree
I didn’t seem to feel any pleasure or relief at all	45.8	47.9	5.6	0.7
I find it difficult to take the initiative to start work	38	50.7	8.8	2.5
I feel like I have nothing to look forward to soon	45.8	44	8.1	2.1
I feel blue and depressed	43.3	49.6	4.9	2.1
I can’t get enthusiastic about anything	55.6	37.3	5.6	1.4
I don’t think I’m good enough to be human	81.7	13	4.2	1.1
I feel that life is meaningless	76.1	18	4.2	1.8

### 3.2 Analysis of correlations between variables

**Figure 2:**
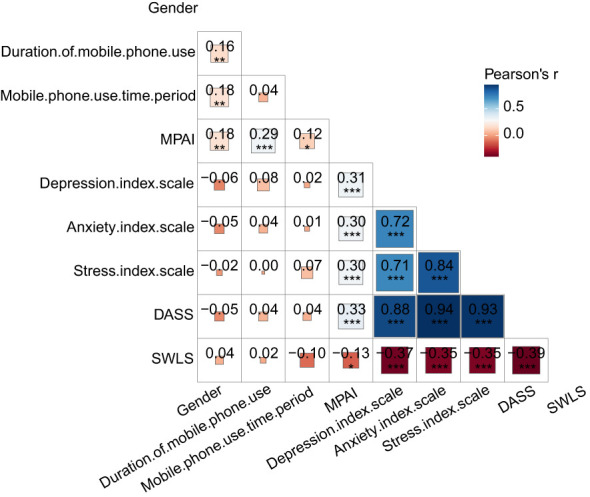
Pearson’s correlations among relevant study variables(N=322), *p<0.05; **p<0.01; ***p<0.001, MPAI: Mobile Phone Addiction Index, DASS: Depression, Anxiety, and Stress Scale, SWLS; Satisfaction with Life Scale.

**Figure 3:**
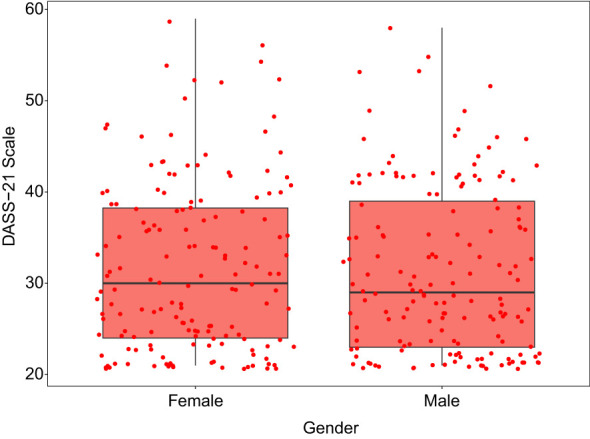
Gender’s score on negative emotion expression.

**Figure 2** reveals that both the time spent on the phone and the duration of phone use are positively correlated with MPAI levels. MPAI levels among university students show a positive correlation with depression, anxiety, and stress (p < 0.01). Conversely, depression, anxiety, and stress levels exhibit a negative correlation with life satisfaction (p < 0.01). Additionally, gender correlates with time spent using the phone, duration of phone use, and phone addiction, with correlation coefficients of r = 0.157, r = 0.180, and r = 0.176, respectively (p < 0.01). Frequent phone users tend to exhibit higher levels of phone addiction, negative emotions, and lower life satisfaction compared to those who use their phones less frequently. Moreover, women score higher on the negative emotions scale compared to men (**Figure 3**).

### 3.3 Mediation testing and path analysis

In order to ensure the accuracy of the mediation effect model and path analysis, considering the huge number of questions, we screened and excluded samples with short response time and basically the same answers. This study employed phone addiction as a control variable, with negative emotions and life satisfaction as mediator and outcome variables, respectively, to conduct mediation effect testing (**Table 4**) and path analysis (**Table 5**). The objective was to explore the mediating role of negative emotions in the relationship between phone addiction and life satisfaction. MPAI was significantly associated with depression (β = 0.094, p < 0.001). The direct effect of MPAI on SWLS was 0.0007, while the indirect effect was -0.6243. Overall, depression largely mediated the relationship between MPAI and SWLS, as indicated by the 95% bootstrap confidence interval of [-0.0639, -0.0083].

**Table 4:** Intermediate effect model.

		effect size	se	LLCI	ULCI
Total effect		-0.625	0.0266	-0.1148	-0.0102
Direct effect		0.0007	0.0262	-0.0509	0.0523
Indirect effect	Depression index	-0.0327	0.0142	-0.0639	-0.0083
	Anxiety index	-0.0141	0.0145	-0.0442	0.0143
	Strees index	-0.0165	0.0163	-0.05	0.0149

**Table 5:** Path analysis.

	R	R2	F	Beta	t	LLCI	ULCI
MPAI→SWLS	0.157	0.025	7.119**	-0.157	-2.668	-1.330	-0.020
MPAI→DS	0.275	0.076	23.111***	0.275	4.807	0.049	0.117
DS→SWLS	0.452	0.205	72.54***	-0.452	-8.517	-0.906	-0.566

MPAI, Mobile Phone Addiction Index; DS, Depression Scale; SWLS, Satisfaction with Life Scale; scale, **p<0.01; ***p<0.001.

It is noteworthy that the direct effect of MPAI on SWLS was not significant in the mediation model (r = 0.0007, p > 0.05), suggesting that phone addiction does not directly affect life satisfaction. However, when negative emotions were included as a mediator, the association between MPAI and SWLS became stronger, reflecting the impact of negative emotional responses. In addition, the experimental results found that there was a strong correlation between mobile phone addiction and depression, anxiety and stress, but only depression and life satisfaction had a significant impact, so only depression was considered as the main line of the study in the path analysis.

**Figure 4:**
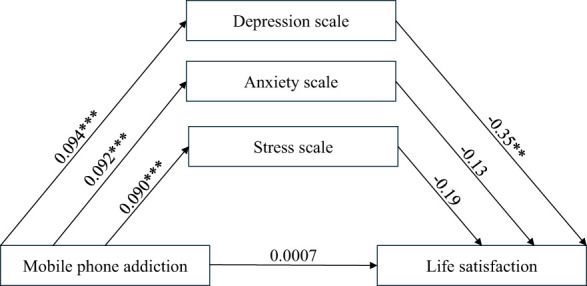
A model of mediating effects between mobile phone addiction, depression, stress, anxiety and life satisfaction.

Path analysis demonstrated a high F-value for the effect of MPAI on depression scales(F = 23.11; p < 0.001), indicating a good fit. Similarly, depression scales had a high F-value for SWLS (F = 72.54; p < 0.001), suggesting a strong fit with the mediation effect model (**Figure 4**). Both models elucidate the role of depression scales in the relationship between MPAI and SWLS, highlighting the significance of managing negative emotions to enhance life satisfaction and mitigate phone addiction.

## 4 Discussion

This study developed a mediation model with mobile phone addiction and negative emotions as predictor and mediator variables, respectively, and life satisfaction as the outcome variable. Mobile phone addiction was conceptualized as excessive use of mobile phones, which affects mood and evolves into negative emotions such as depression and anxiety. This phenomenon can be understood in terms of cognitive processes and the expression of negative emotions.

### 4.1 Influence of habitual use on mobile phone addiction across different demographic and sociological characteristics

The study identified habitual use as a significant determinant of mobile phone addiction. The isolation policies implemented to prevent the spread of the epidemic (e.g., working and studying from home) increased mobile device usage, thereby heightening the risk of addiction among university students (43). Additionally, the large amount of free time during home confinement revealed an increase in both the duration and frequency of mobile phone use, which are indicative of addiction (52). Descriptive statistics and correlation analysis demonstrated that the duration and frequency of mobile phone use are significantly associated with mobile phone addiction (**Figure 2**; p < 0.01). Thus, a higher degree of habitual phone use is linked to increased duration and frequency of use, contributing to addiction.

The study also found a significant correlation between gender and mobile phone addiction (**Figure 2**; p < 0.01), with females more likely than males to express negative emotions (**Figure 3**). Previous research indicates that girls tend to be more confrontational when expressing negative emotions, which can exacerbate their own emotional distress (53). While boys’ mobile phone addiction might be related to online gaming, girls often exhibit a greater need for social communication and online shopping (54–56). Girls are particularly vulnerable to addiction due to their heightened social needs and stress (57). Additionally, mobile phone addicts often require increased intensity and duration of use to mask their negative emotions, with females being particularly susceptible to this phenomenon (29). Consequently, the significant rise in mobile phone use and the ease with which females can become deeply immersed in the digital world have made them more vulnerable to addiction.

### 4.2 Impact of mobile phone addiction on negative emotions

Individuals with smartphone addiction frequently experience reduced life satisfaction and well-being due to heightened negative emotions (**Table 4**). Previous studies have also shown that cognitive habits, academic stress, and social stress affect life satisfaction (58–60). Mobile phone addiction can create a detrimental cycle where negative emotions drive increased phone use for social activities, which in turn worsens negative emotions due to prolonged engagement in phone-based social interactions (61, 62). Hawi and Samaha observed a positive correlation between mobile phone addiction and depression (63). Thus, mobile phone addiction amplifies negative emotions by disrupting cognitive control.This study found a significant correlation between high levels of mobile phone addiction and increased negative affect, including a notable correlation between mobile phone addiction and depression, as well as between depression and life satisfaction (**Table 5**). Specifically, the relationship between mobile phone addiction and depression is positive (**Figure 2**; p < 0.01, r = 0.307), whereas the relationship between depression and life satisfaction is negative (**Figure 2**; p < 0.01, r = -0.372). Depression, a prevalent mental health issue, not only impacts emotional and cognitive needs but also impairs overall well-being and perception of the external world (64). Emotional interference and cognitive control can influence depressive symptoms (65). Therefore, individuals with higher levels of mobile phone addiction are more prone to emotional interference and impaired cognitive control, resulting in increased negative emotions and a substantial decline in life satisfaction.

### 4.3 Impact of negative emotions on life satisfaction

The study revealed a significant negative correlation between negative emotions and life satisfaction (**Figure 2**; p < 0.01, r = -0.391). Mobile phones offer a virtual environment where users can find information, resources, and express negative emotions. Previous research suggests that smartphone use may serve as a strategy for experiential avoidance, helping individuals evade aversive emotional content (66). For individuals with depressive tendencies, online social resources can alleviate psychological burden (67). However, self-indulgence during smartphone use can increase addiction propensity (68). According to compensatory internet theory, smartphones may help college students relieve psychological stress while simultaneously increasing addiction tendencies. Once addiction to mobile phones occurs, emotional instability can lead to negative attitudes toward external events (69). Accumulated depression can intensify the need for addiction, further worsening depressive symptoms. As for the significance of depression, anxiety, stress and life satisfaction, it can be inferred that in college students, learning pressure, life pressure and various kinds of anxiety are everywhere, but these cannot directly change life satisfaction. Depression is a kind of mental disease, which can lead to the reduction of people’s communication ability and thinking mode, thus causing a significant impact on life satisfaction.

**Figure 5:**
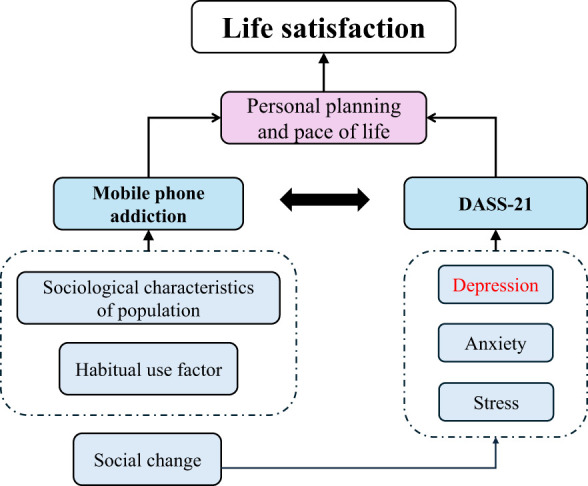
Structure frame diagram for discussion.

Overall, while the Internet has provided significant convenience, habitual mobile phone use can lead to numerous negative emotions. Chinese college students, facing societal transitions and pressures, may experience increased anxiety and depression. This often results in using mobile phones to avoid reality, exacerbating addiction. Consequently, students may become immersed in negative emotions, leading to lower life satisfaction (**Figure 5**).

## 5 Implications and recommendations

This study offers valuable theoretical insights into the relationship between mobile phone addiction and life satisfaction. It demonstrates that mobile phone addiction adversely affects life satisfaction. To mitigate mobile phone addiction, educational institutions should consider several strategies. They can invest in modern sports facilities, organize diverse campus activities, and foster enhanced communication and collaboration among students. These measures could help reduce dependency on mobile phones. Furthermore, this study introduces emotional changes as a mediating factor and establishes a mediation model demonstrating how mobile phone addiction negatively impacts life satisfaction through negative emotions. Excessive use of mobile phones is linked to the accumulation of negative emotions, such as depression and anxiety, which in turn diminish life satisfaction. To address this, schools should implement psychological support services, including counseling stations, to monitor students’ mental health and assist them with psychological issues. Students should also engage in proactive communication with their peers and manage negative emotions effectively to lower the risk of depression.

## 6 Limitations and shortcomings

This study has several limitations. First, while it effectively demonstrates the moderating role of mobile phone addiction on life satisfaction, the sample used was not sufficiently representative. The research primarily focused on undergraduate students, which may limit the generalizability of the findings to the broader college student population. Second, factors such as depression, stress, and anxiety can be influenced by numerous variables not explored in this study. Additionally, the study did not provide a comprehensive analysis of the underlying mechanisms of these psychological states, which warrants further investigation in future research.

## Data Availability

The original contributions presented in the study are included in the article/supplementary material. Further inquiries can be directed to the corresponding author.
